# Inducible forebrain-specific ablation of the transcription factor *Creb* during adulthood induces anxiety but no spatial/contextual learning deficits

**DOI:** 10.3389/fnbeh.2014.00407

**Published:** 2014-11-27

**Authors:** Miriam A. Vogt, Dragos Inta, Alessia Luoni, Hasan Elkin, Natascha Pfeiffer, Marco A. Riva, Peter Gass

**Affiliations:** ^1^Department of Psychiatry and Psychotherapy, RG Animal Models in Psychiatry, Central Institute of Mental Health, Medical Faculty Mannheim / Heidelberg UniversityMannheim, Germany; ^2^Department of Pharmacological and Biomolecular SciencesUniversity of Milan, Milan, Italy

**Keywords:** mouse, CREB, anxiety, tamoxifen induction, learning, memory, CREM, BDNF

## Abstract

The cyclic AMP (cAMP)-response element binding protein (CREB) is an activity-dependent transcription factor playing a role in synaptic plasticity, learning and memory, and emotional behavior. However, the impact of *Creb* ablation on rodent behavior is vague as e.g., memory performance of different *Creb* mutant mice depends on the specific type of mutation *per se* but additionally on the background and learning protocol differences. Here we present the first targeted ablation of CREB induced during adulthood selectively in principal forebrain neurons in a pure background strain of C57BL/6 mice. All hippocampal principal neurons exhibited lack of CREB expression. Mutant mice showed a severe anxiety phenotype in the openfield and novel object exploration test as well as in the Dark-Light Box Test, but unaltered hippocampus-dependent long-term memory in the Morris water maze and in context dependent fear conditioning. On the molecular level, CREB ablation led to CREM up regulation in the hippocampus and frontal cortex which may at least in part compensate for the loss of CREB. BDNF, a postulated CREB target gene, was down regulated in the frontal lobe but not in the hippocampus; neurogenesis remained unaltered. Our data indicate that in the adult mouse forebrain the late onset of CREB ablation can, in case of memory functionality, be compensated for and is not essential for memory consolidation and retrieval during adulthood. In contrast, the presence of CREB protein during adulthood seems to be pivotal for the regulation of emotional behavior.

## Introduction

Inhibition of transcription factors or protein synthesis is hypothesized to block consolidation of short-term memory into long-term memory and affect emotional and depression-related circuits. The role of cAMP signaling in simple forms of learning and memory was described for the first time in the sea snail *Aplysia* (Brunelli et al., 1976), a result confirmed by comparable results in *Drosophila* some years later (Byers et al., 1981; Yin et al., 1994). To date, a multitude of studies revealed CREB, the cAMP-response element binding protein as the main element in converting short- to long-term memory (Barco et al., 2003; Kim et al., 2013). Apart from its role in learning and memory, CREB is known to modify the sensitivity to rewarding and aversive drugs within the Nucleus accumbens (Dinieri et al., 2009; Bilbao et al., 2014), resets the circadian clock by its phosphorylation at SER142 (Gau et al., 2002) and is upregulated in the hippocampus by chronic antidepressant treatment, linking *Creb* activity to the pathogenesis and therapy of depression and regulation of emotion (Gass and Riva, 2007).

CREB is part of the family of activating transcription factors including besides CREB the cAMP responsive element modulator (CREM) and the activating transcription factors 1–7 (ATF; Brindle and Montminy, 1992). Due to the complex structure of the *Creb* gene with multiple exons and introns, the encoded proteins vary with splicing variants and different properties (Blendy et al., 1996; Mayr and Montminy, 2001). This creates a wide range of possibilities of genetic approaches to modify expression, formation or function of *Creb* (for an overview see Kida and Serita, 2014). The homozygous null mutation of *Creb* is perinatally lethal, therefore no behavioral analysis is available, the mice suffer from apoptosis and degeneration of sensory neurons combined with reduced axonal growth and projections (Rudolph et al., 1998; Lonze et al., 2002). The development of temporal and spatially restricted mutants using the Cre/loxP system allowed generating mutant mice with CREB ablation in forebrain neurons only (Mantamadiotis et al., 2002), however, this line was developed on a Crem negative background to avoid compensatory effects of Crem regulation, although it is known that Crem ablation leads to altered emotional behavior and hyperactivity (Maldonado et al., 1999). Behavioral phenotyping aiming on emotional regulation was mostly focusing on depression-related changes, although *Creb*αΔ mice, which carry a constitutive deletion of the α and Δ isoforms of CREB, show baseline alterations in anxiety-like behavior (Conti et al., 2002). In humans, a rare genomic variant could link bipolar disorder including co-morbid anxiety to intracellular pathways under the regulation of *Creb* (Kerner et al., 2013). Mice with virus-mediated hippocampal *Creb* ablation demonstrate no baseline anxiety alterations but enhanced neurogenesis, which could lead in longer time frames to emotional changes (Gundersen et al., 2013), an aspect which we wanted to evaluate in our mice by assessing adult neurogenesis. Learning and memory tasks were conducted for the first time 1992 in mice generated by using the tetracycline-controlled transactivator/operator system expressing a dominant negative inhibitor of *Creb* (Walton et al., 1992) active in CamKIIα-positive cells of the forebrain only. As predicted from studies with invertebrates, long-term memory but not short-term memory was impaired, although the mutant mice exhibited no contextual fear conditioning deficit (Pittenger et al., 2002). The blockade of *Creb* using a tamoxifen-inducible expression of a dominant negative *Creb* repressor was used by Kida et al. (2002) to dissect the crucial role of CREB in consolidation of contextual fear conditioning.

Even though a plethora of mice with alterations of *Creb* expression or CREB function is available (Kida et al., 2002), the behavioral phenotype has not been consistent across lines. The direct comparison of the multiple studies was aggravated by the fact that the mouse lines differ in their genetic system, e.g., if the line was generated with partial gene deletions as the *Creb*αΔ mice (Bourtchuladze et al., 1994) tissue specific deletions as deletions only in the forebrain (Pittenger et al., 2002), time-dependent induction (Kida et al., 2002), or if additional genes were knocked down to avoid compensatory effects e.g., of Crem (Mantamadiotis et al., 2002). While studies about emotional behavior regarding anxiety were almost missing, learning performance was, already within single lines, dependent on protocol (with or without spaces between trials in the Morris water maze) or background strains. We have chosen here the inducible Cre/loxP recombination system with tamoxifen-controlled gene manipulation in mice on a pure C57BL/6 background suitable for behavioral phenotyping. *Creb* ablation was induced during adulthood to analyze emotional behavior, learning and memory and additionally, mRNA levels of CREM, ATF-1 and BDNF in hippocampus and frontal cortex.

## Materials and methods

### Generation of Crebflox/flox^CamKCreERT2^ mice and induction of Cre-mediated recombination with tamoxifen

Transgenic mice expressing the tamoxifen-inducible fusion protein composed of the Cre recombinase and the mutated ligand binding domain of the human estrogen receptor under the control of the αCamKII promoter (CamKCreER^T2^; Erdmann et al., 2007), were crossed with homozygous Creb1flox/flox mice (Mantamadiotis et al., 2002) to generate Crebflox/flox^CamKCreERT2^ mice. Systemic tamoxifen injection to all mice led to the excision of the exon 10 of the *Creb* *1* allele in excitatory neurons of the forebrain region including hippocampus, amygdala, cortex and striatum only in mutant mice. Crebflox/flox^CamKCreERT2^ mice were treated with tamoxifen at the age of 9–12 weeks. Tamoxifen (Sigma, Deisenhofen, Germany) was dissolved in ethanol absolute (100 mg tamoxifen per 1 ml ethanol) and then diluted 1:10 with sunflower seed oil (Sigma). Crebflox/flox^CamKCreERT2^ mice (“mutants”) and Crebflox/flox mice (“controls”) were injected intraperitoneally twice a day with 100 μl (i.e., 1 mg) tamoxifen, for 5 days. All mouse lines were bred for at least 10 generations on a C57BL6/N background.

### Behavioral experiments

The behavioral testing started when the animals were approximately 5 months old. Only male mice were tested. Two weeks prior to and during experiments, animals were single-housed in a 12 h reversed dark-light cycle with lights on at 7 pm and supplied with food and water ad libitum. All experiments were performed during the dark phase. All genotypes were verified with immunohistochemistry controlling for CREB ablation. All experimental procedures were approved by the Animal Welfare Committee (Regierungspräsidium Karlsruhe, 35-9185-81-G/193/11) and carried out according to the European Communities Council Directive of 24 November 1986 (86/609/EEC). One month after termination of the behavioral experiments, *n* = 12 mice per genotype, respectively, received a intraperitoneal injection of BrdU (50 mg/kg) and were sacrificed 1 day later to evaluate neurogenesis.

#### Openfield and novel object exploration test

Activity monitoring was conducted in a square shaped, white open field, measuring 50 × 50 cm^2^ and illuminated from above by 25 lx. Mice were placed individually into the arena and monitored for 20 min by a Video camera (Sony CCD IRIS). The resulting data were analyzed using the image processing system EthoVision 3.1 (Noldus Information Technology, Wageningen, the Netherlands). Parameters assessed were total distance moved, velocity, and time in center, which was defined as the area 10 cm distant from the walls. After 10 min Open field Test, a novel object was introduced into the middle of the arena. Object exploration was assessed for the subsequent 10 min, assessing latency of first approach, and the total number of approaches (*N* = 24 per genotype) as previously described (Berkel et al., 2012).

#### Dark-Light Box

The Dark-Light-Box consisted of two plastic chambers, connected by a small tunnel. The dark chamber measured 20 × 15 cm^2^ and was covered by a lid. The adjacent chamber, measuring 30 × 15 cm^2^, was white and illuminated from above by 600 Lux. Mice were placed into the dark compartment and latency to first exit, number of exits and total time in the light compartment were recorded for 5 min (*N* = 24 per genotype) as described earlier (Fuss et al., 2010).

#### Contextual fear conditioning

For the contextual conditioning, mice were individually placed into the conditioning chamber (58 × 30 × 27 cm^3^, TSE, Bad Homburg, Germany) and allowed to habituate for 2 min before subjecting them to the unconditioned stimulus (2 s of continuous footshock of 0.8 mA). 24 h after training, context conditioning was assessed by measuring freezing, defined as a complete lack of movements apart from respiration. Context learning was tested in the same conditioning chamber which was used during the training. Freezing behavior was scored manually at intervals of 10 s for 5 min (*N* = 14 Crebflox/flox^CamKCreERT2^ mutant mice, *n* = 10 controls) as described earlier (Fleischmann et al., 2003).

#### Morris water maze

Animals (*n* = 14 controls, *n* = 10 mutants) were trained for 4 days with a total of 24 trials (6 trials per day) to swim in a water-filled circular pool (diameter: 150 cm) and find a platform (14 × 14 cm^2^, plexiglas) (acquisition phase). In each swim trial during the acquisition phase, in which the position of the platform was kept unchanged, mice were left in the pool for a maximum of 120 s or they found the platform. On the fifth day the platform was removed and the mouse was recorded for 60 s (probe trial). With a video camera suspended above the center of the pool, the swim tracks of the mice were analyzed using the image processing system EthoVision X8 (Noldus Information Technology, Wageningen, the Netherlands). The following variables from the recorded paths were analyzed: time to find platform (s), length of swim path (m), velocity (cm/s), percent of time spent moving, percent of time spent within a rim of 20 cm from the wall, in a ring in which all 4 possible platform positions are included and additionally in the center. For the probe trial, additionally percent of time in target and other quadrants, as well as number of crossings of the former platform area in comparison to 3 other possible platform positions were analyzed as formerly described (Vogt et al., 2008). Animals showing movement less than 80% of the total time (mean over all acquisition trials and the probe trial) were excluded from the analysis of the experiment, which resulted in *N* = 8 controls and *N* = 7 mutants.

#### Hotplate Test

The mice were tested on the hotplate test (ATLab, Vendargues, France). Temperature was set at 53°C (±0.3°C) and a 45 s cut-off was determined to prevent injury of mice. Latency to first reaction, i.e., licking hind paws or jumping, was assessed (*N* = 24 per genotype) as described elsewhere (Chourbaji et al., 2008).

### Immunohistochemistry

One day after the BrdU injection all animals were perfused transcardially with 4% paraformaldehyde (PFA), brains were postfixed overnight and 40 μm thick coronal sections were cut on a vibratome as described elsewhere (Böttiger et al., 1999). Every sixth section of each animal was processed free-floating.

Dividing cells were visualized using primary rat monoclonal anti-BrdU-antibody (1:1000, MAK 2060, Linaris, Wertheim-Bettingen, Germany). To determine the absolute number of BrdU-labeled cells, we used the peroxidase method (ABC system, Vectastain, Vector Laboratories, Burlingame, CA, USA) with biotinylated anti-rat antibodies (1:500; Dianova, Hamburg, Germany). Nickel-intensified diaminobenzidine (DAB, Sigma) was used as chromogen substrate (Römer et al., 2010).

CREB expression was analyzed by incubating the sections overnight with polyclonal rabbit anti-CREB antibody (1:50000, Cell Signaling). After washes with PBST, sections were incubated with secondary antibody (CREB: biotinylated anti-rabbit IgG, Vector laboratories), 1:400 for 2 h at room temperature. After washes, sections were processed with avidin-biotinylated horseradish peroxidase complex (Vectastain ABC kit; Vector Laboratories) in PBST for 1 h at RT, and the reaction was visualized using Nickel-3,30-diaminobenzidine (DAB; Strekalova et al., 2003).

### Cell counts

To evaluate proliferation, the total number of BrdU-positive cells was assessed in 1-in-6 series of sections (240 μm apart) from all animals. BrdU-positive cells were counted throughout the rostro-caudal extent of the granule cell layer (GCL) using a 40 × objective. The optical dissector method was modified as described previously, in that cells appearing in the uppermost focal plane, when focusing into the section, were not counted. The resulting numbers were then multiplied by six to obtain the estimated total cell number (*N* = 12 per genotype).

### RNA Preparation for qRT-PCR and analysis of mRNA levels

Naïve male mice not used for behavioral analyses (*N* = 12 per genotype) were killed by cervical dislocation and the brain was quickly removed. Hippocampus and frontal cortex were dissected on ice, immediately frozen on dry ice and stored by −80°C until further analyses. Total RNA was isolated by single step guanidinium isothiocyanate/phenol extraction using PureZol RNA isolation reagent (Bio-Rad Laboratories, Italy), according with the manufacturer’s instructions and quantified by spectrophotometric analysis. Following total RNA extraction, the samples were processed for real-time polymerase chain reaction (qPCR) to assess mRNA levels. An aliquot of each sample was treated with DNase to avoid DNA contamination.

RNA was analyzed by TaqMan qRT-PCR instrument (CFX384 real time system, Bio-Rad Laboratories) using the iScriptTM one-step RT-PCR kit for probes (Bio-Rad Laboratories). Samples were run in 384 well formats in triplicate as multiplexed reactions with a normalizing internal control (*36B4*) as described elsewhere (Chourbaji et al., 2012).

TaqMan gene expression assays were purchased from Eurofins MWG-Operon (Germany) and have the following sequences:

**Table:** 

Crem	forward primer: TTTCCTCTGATGTGCCTGGT, reverse primer: CCCGTGCTAGTCTGATATATGC, probe: CCACCTAACATTGCTACCATGG;
Atf-1	forward primer: TGAAGATACACGGGGCAGAA, reverse primer: ATGGCAATGTACTGTCCGCT, probe: GCATTTCTGCCATCACGTCT;
total Bdnf	forward primer: AAGTCTGCATTACATTCCTCGA, reverse primer: GTTTTCTGAAAGAGGGACAGTTTAT, probe: TGTGGTTTGTTGCCGTTGCCAAG;
36B4	forward primer: AGATGCAGCAGATCCGCAT, reverse primer: GTTCTTGCCCATCAGCACC, probe: CGCTCCGAGGGAAGGCCG.

Thermal cycling was initiated with an incubation at 50°C for 10 min (RNA retrotranscription) and then at 95°C for 5 min (TaqMan polymerase activation). After this initial step, 39 cycles of PCR were performed. Each PCR cycle consisted of heating the samples at 95°C for 10 s to enable the melting process and then for 30 s at 60°C for the annealing and extension reaction. Relative target gene expression was calculated according to the 2(-Delta Delta C(T)) method.

### Statistical analysis

Statistical analyses for behavioral tests, the mRNA levels and the cell counts were performed using SPSS Statistics 20 for Windows. Inter-group comparisons were calculated by Student’s *t*-tests. Where appropriate, the model was complemented by within subject factors to explore the dependence of genotype effects on time (data from acquisition phase of the Morris Water Maze). Significance for all tests was assumed for *p* < 0.05. Data are presented as means ± standard error (S.E.M.).

## Results

**Figure 1:**
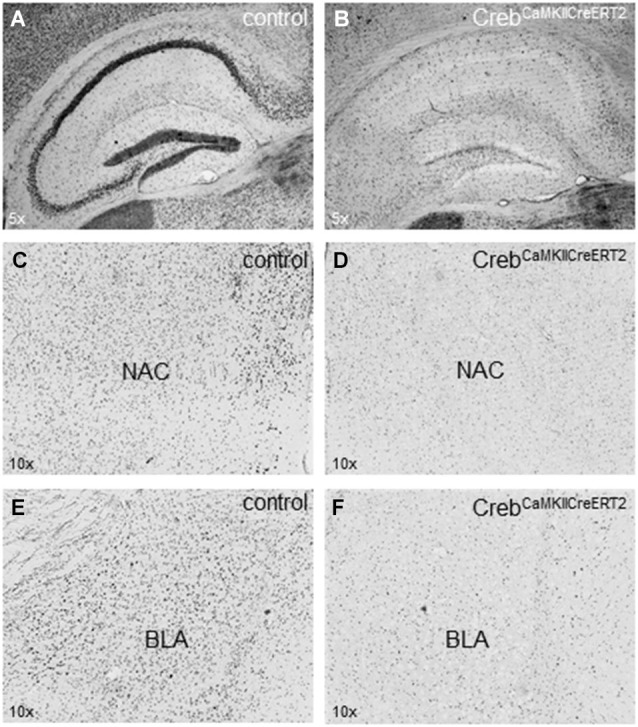
**Expression of CREB in hippocampus, nucleus accumbens and amygdala. (A)** Hippocampal expression of CREB in (tamoxifen-treated) control animals with regular CREB expression and **(B)** in Creb^CamKCreERT2^ mutant mice after induced ablation with tamoxifen in the adulthood with a definite loss of CREB expression in the pyramidal cell layer but not in putative GABAergic interneurons sparsely distributed in all hippocampal layers. **(C)** Expression of CREB in the nucleus accumbens (NAC) important for rewarding mechanisms in (tamoxifen-treated) control animals with regular CREB expression and **(D)** in Creb^CamKCreERT2^ mutant mice after induced ablation with tamoxifen in the adulthood. **(E)** Expression of CREB in the basolateral amygdala (BLA) important for fear-related memory in control and **(F)** in Creb^CamKCreERT2^ mutant mice after induced ablation with tamoxifen in the adulthood. *N* = 12 per genotype.

Crebflox/flox^CamKCreERT2^ mice were treated with tamoxifen at the age of 9–12 weeks (adulthood), which resulted in the ablation of CREB in excitatory forebrain neurons including all principal neurons of the hippocampus (Figures 1A,B). We detected a reduction of CREB expression in additional regions of the limbic system important e.g., for reward mechanisms (nucleus accumbens, Figures 1C,D) and for fear memory (basolateral amygdala, Figures 1E,F).

Mice with a CREB ablation during adulthood thrived well and did not show defects concerning body growth or bodyweight after induction and especially during testing, and gained weight normally (repeated measurement ANOVA after onset of treatment: F_time_(3,66) = 39,197 *p* < 0.001).

**Figure 2:**
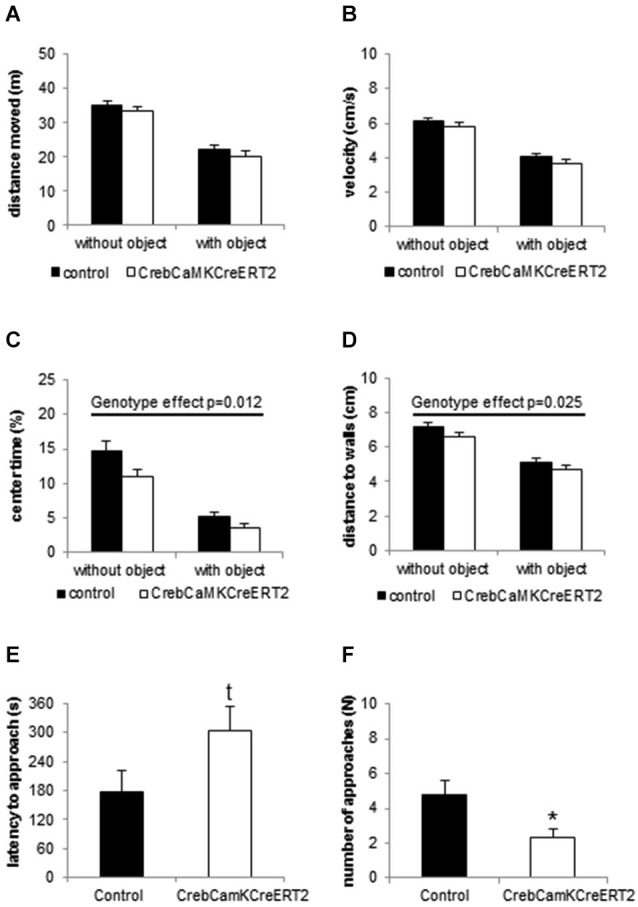
**Locomotion and Novel object exploration in the openfield. (A)** Distance moved and **(B)** velocity is unaltered in Creb^CamKCreERT2^ mutant mice compared to controls in the openfield without object (first 10 min) and after introduction of the novel object. **(C)** Center time and **(D)** mean distance to walls is reduced in Creb^CamKCreERT2^ mutant mice, reflecting anxiety-like behavior. **(E)** Latency to approach the novel object is enhanced (*p* = 0.059) and **(F)** the number of approaches is decreased in Creb^CamKCreERT2^ mutant mice compared to controls. Black bars: controls, white bars: Creb^CamKCreERT2^ mutant mice, * *p* < 0.05, *N* = 24 per genotype.

In the openfield and novel object exploration test, Crebflox/flox^CamKCreERT2^ mice, when compared to controls, did not show locomotor disturbances with regard to the distance traveled and velocity (Figures 2A,B). Concerning the spatial pattern of movement, Crebflox/flox^CamKCreERT2^ mutant mice displayed reduced time in center and distance from the nearest wall (Figures 2C,D; repeated measurement ANOVA center time: F_genotype_(1,42) = 6.814 *p* = 0.012; distance to walls: F_genotype_(1,42) = 5.429 *p* = 0.025), reflecting an anxiety-like behavior, accompanied by an increased latency to explore the novel object (Figure 2E; student *t*-test: *p* = 0.059) and reduced number of approaches (Figure 2F; *p* = 0.017).

**Figure 3:**
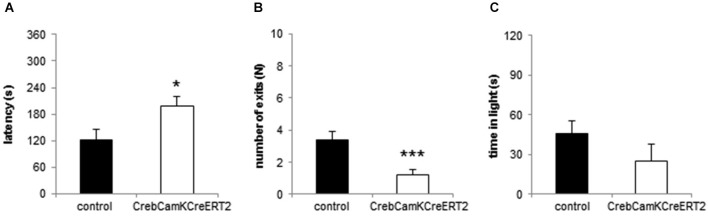
**Anxiety-like behavior in the Dark-Light Box. (A)** The latency to enter the light compartment is enhanced and the **(B)** number of exits is reduced in Creb^CamKCreERT2^ mutant mice compared to controls. **(C)** Time in the bright compartment is unaltered in Creb^CamKCreERT2^ mutant mice compared to controls. Black bars: controls, white bars: Creb^CamKCreERT2^ mutant mice, * *p* < 0.05, *** *p* < 0.001, *N* = 24 per genotype.

In the Dark-Light Box, Crebflox/flox^CamKCreERT2^ mutant mice demonstrated anxiety-like behavior by entering the bright compartment with a significantly increased latency (Figure 3A; *p* = 0.019), displayed significantly less exits to the bright compartment (Figure 3B; *p* < 0.001) and stayed less (but non-significantly) in the light (Figure 3C; *p* = 0.193).

**Figure 4:**
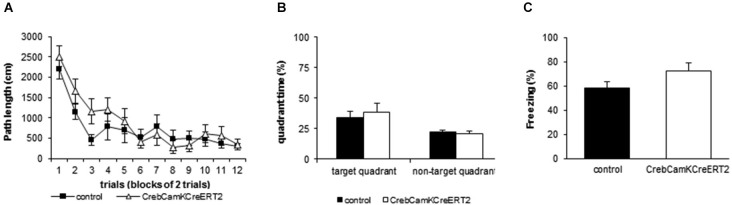
**Learning and Memory performance in Morris water maze and contextual fear conditioning. (A)** Mutant mice with a loss of *Creb* in the adulthood do not show disturbed learning during acquisition in the Morris water maze as represented by the distance moved to reach the platform (repeated measurement ANOVA factor time: F_(11,132)_ = 18.975, *p* < 0.001). All data points represent the mean (±SEM), *N* = 8 controls and *N* = 7 mutants. **(B)** Induced loss of *Creb* in the adulthood does not affect spatial reference memory in the Morris water maze as represented by the time spent in the target zone vs. the means of the 3 other quadrants during the probe trial (two-way ANOVA factor place: F_(1,13)_ = 19.122, *p* < 0.001, factor genotype: F_(1,13)_ = 1.752, *p* = 0.208). All data points represent the mean (+SEM), *N* = 8 controls and *N* = 7 mutants. **(C)** Induced loss of *Creb* in the adulthood does not affect hippocampus-dependent associative learning in the fear conditioning paradigm. All data points represent the mean (+SEM) percent time spent freezing during context replacement 24 h after training. *N* = 14 controls, *N* = 10 mutants.

In the Morris water maze, irrespective of the genotype, all mice demonstrated an improved path length (Figure 4A) and a diminished latency to find the platform over time (repeated measurement ANOVA: path length F_time_(11,143) = 21.441 *p* < 0.001, latency to find platform: F_time_(11,143) = 9.674 *p* < 0.001, velocity: F_time_(11,143) = 6.732 *p* < 0.001). Concerning the spatial swimming patterns, both groups focused their swimming to the relevant areas of the pool and decreased wall time and the time in the center over trials (repeated measurement ANOVA: Time in center: F_time_(11,143) = 2.232 *p* = 0.016, Time in wall zone: F_time_(11,143) = 6.203 *p* < 0.001, Time in platform ring zone: F_time_(11,143) = 5.050 *p* < 0.001). No significant differences between *Creb* mutant mice and the control group were found for all measured parameters including velocity and spatial swimming pattern in different zones of the pool, including wall zone and center. During the probe trial, both groups exhibited a significant preference for the trained platform position, measured as time spent in the target quadrant (Figure 4B) and crossings above the previous platform position (two-way ANOVA: time in quadrant: F_place_(1,13) = 7.093 *p* = 0.020, crossings F_place_(1,13) = 11.122 *p* = 0.005). Both *Creb* mutant mice and controls preferred the target quadrant to the same extent and showed comparable number of crossings (two-way ANOVA: time in quadrant: F_genotype_(1,13) = 0.238 *p* = 0.634, crossings F_genotype_(1,13) = 1.445 *p* = 0.251).

Pain perception, measured as latency to the first reaction on the hotplate (licking hind paws or jumping), was unaltered in *Creb* mutant mice (controls: 23.30 ±2.02 s; mutants: 23.67 ±2.61 s).

In the Fear Conditioning paradigm, mice were exposed to a single foot shock (0.8 mA) in a certain context. When replaced in this context 24 h later, all mice showed context memory to the same extent as measured by the time spent freezing (Figure 4C; controls: 58.10 ± 5.3%; mutants: 72.08 ± 6.9%).

**Figure 5:**
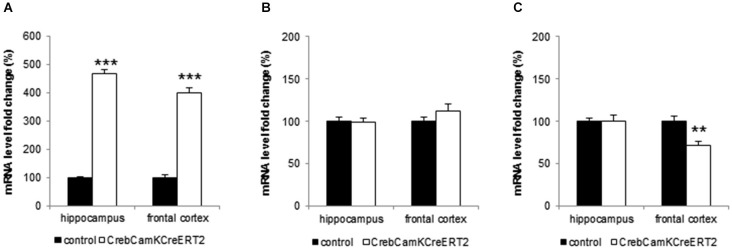
**Analysis of CREM, ATF-1 and BDNF- mRNA level in hippocampus and frontal cortex. (A)** Mutant mice with a loss of *Creb* at adulthood exhibit significant higher mRNA levels of CREM in the hippocampus and frontal cortex. **(B)** Induced loss of *Creb* did not affect ATF-1 mRNA level in hippocampus and frontal cortex. **(C)** Mutant mice express significantly less BDNF mRNA in the frontal cortex but its levels remain unchanged in the hippocampus. All data points represent mean (+SEM), stated as fold change of the control level. Black bars: controls, white bars: Creb^CamKCreERT2^ mutant mice, ** *p* < 0.01, *** *p* < 0.001, *N* = 12 per genotype.

Crem, ATF-1 and BDNF mRNA level were analyzed in the hippocampus and the frontal cortex of naïve male mice. Crem mRNA level were significantly up regulated in Crebflox/flox^CamKCreERT2^ mutant mice when compared to control animals (Figure 5A; student *t*-tests: hippocampus: *p* < 0.001, frontal cortex *p* < 0.001). Conversely, ATF-1 level remained unaltered in both hippocampus and frontal cortex (Figure 5B). In contrast, BDNF mRNA level were slightly, although significantly, decreased in the frontal cortex of mutant mice (Figure 5C; *p* = 0.0017), whereas its expression was unchanged in the hippocampus.

Proliferation, measured as number of BrdU-positive cells, was unaltered in Crebflox/floxCamKCreERT2 mice (data not shown).

## Discussion

We used an improved Cre/loxP recombination system with tamoxifen-controlled gene manipulation, for time- and region-restricted deletion of *Creb* (Erdmann et al., 2007). Gene ablation was induced at early adulthood (9–12 weeks) to avoid vulnerable phases as puberty and especially to exclude adaptations through compensatory mechanisms affecting neurodevelopment (Aguado et al., 2009; Nonaka, 2009). All animals were treated with tamoxifen to exclude treatment effects by tamoxifen, which had been confirmed in wild-type mice in particular in depression-related tasks (Vogt et al., 2008). The tamoxifen treatment led to an ablation of CREB protein in excitatory forebrain neurons (including hippocampus, nucleus accumbens and basolateral amygdala) expressing regulatory elements of the CamKIIα gene. The induction and the subsequent ablation of the CREB protein in tamoxifen-injected Crebflox/flox^CamKCreERT2^ mice occurred within a few days, as described in detail for the glucocorticoid receptor (GR) by Erdmann et al. (2007). The tamoxifen injections led to an expression pattern comparable to those described by Gundersen et al. (2013), where Crebflox/flox mice were injected with AAV-Cre virus in the hippocampus to induce the deletion.

To dissect anxiety-like behavior the animals underwent an openfield and Novel object exploration task as well as the Dark-Light Box test, classical approach- avoidance conflict tasks. Whereas locomotion was not altered, *Creb* mutant mice avoided the central part of the arena, and approached a novel object placed in the middle later and less often. The Dark-Light Box test revealed correspondingly higher anxiety levels in our Crebflox/flox^CamKCreERT2^ mice.

Several studies have shown that altered levels of CREB or pCREB, the phosphorylated active form of CREB, affect anxiety levels directly or indirectly. Mice with early onset of *Creb* ablation throughout the nervous system (Creb^NesCre^ mice) and CrebαΔ knock-out mice with a hypomorphic allele and highly reduced CREB levels show in accordance with the present data higher anxiety levels in elevated zero maze, elevated plus maze Dark-Light Box and openfield (Valverde et al., 2004; Gur et al., 2007). Virus-mediated expression of a dominant negative form of CREB increased anxiety-like behavior, however in this case the target of gene ablation was the Nucleus accumbens shell only (Barrot et al., 2002), an effect which could be reversed by anxiolytic drug treatment or overexpression of *Creb* in the Nucleus accumbens (Barrot et al., 2005). In the mice used in this study, the nucleus accumbens was affected by the reduction of CREB levels, but it is rather unlikely that the complex anxiety phenotype found here is based on one affected brain structure as the shell of the Nucleus accumbens, although the Nucleus accumbens might be a crucial brain structure. Chronic social defeat reduced CREB and BDNF levels in the raphe, and increased in parallel anxiety levels (Boyarskikh et al., 2013). The knockdown of the corticotropin-releasing hormone receptor 2 (CRH2), known to phosphorylate CREB, resulted, as expected, in reduced pCREB and, additionally, higher anxiety levels (Kishimoto et al., 2000). Reduced pCREB levels via indirect effects combining several studies from different fields of neuroscience all resulted in increased anxiety levels analyzed in standard test of anxiety as openfield, elevated plus maze and/or Dark-Light Box test (for review of altered anxiety in CREB-, BDNF- and CRH1-mutant mice see also Urani et al., 2005).

Animal studies aiming on Alzheimers’ disease either focusing on the enzyme 12/15–lipoxygenase catalyzing the deoxygenation of polyunsaturated fatty acids, or focusing on the carboxy terminal fragments of the human amyloid precursor protein (betaCTF99) revealed CREB alterations and corresponding anxiety changes (Lee et al., 2006; Joshi et al., 2014). Correspondingly, the upregulation of *Creb* expression or pCREB levels was followed by reduced anxiety in studies using pharmacological approaches or knockout mice. Thus, the knockdown of the regulator of calcineurin 1 (RCAN1)—a protein regulating the calcium/calmodulin-dependent phosphatase calcineurin implicated in human anxiety disorders—increased in mice pCREB and BDNF levels. Moreover, these mice displayed innate reduced anxiety levels, an effect which could not be reversed by treatment with the selective serotonin reuptake inhibitor (SSRI) fluoxetine (Hoeffer et al., 2013). Interestingly, Mombereau et al. (2010) showed in *Creb*αΔ knock-out mice that Citalopram, another SSRI, could only be effective (and consequently decrease anxiety) when the animals expressed normal CREB levels. Serotonergic pathways driving not only SSRI response but furthermore CREB signaling were known to be affected as shown by Stewart et al. (2014). In this study, the endogenous regulator of G-protein signaling 6 (RGS6) implicated as inhibitor of 5-HT_1a_ receptors, was knocked out which resulted in anxiety and pCREB level alterations. Interrelations of 5HT_1a_ and *Creb* are furthermore demonstrated by Mombereau et al. (2010) who could show that *Creb*αΔ mice react with a blunted response in the 5-HT_1a_ agonist-induced hypothermia test following a single administration of 8-OHDPAT.

BDNF levels appear to play an important role in *Creb*-dependent regulation of emotional behavior, since peripherally administered BDNF was observed to increase pCREB and, in parallel, to reduce anxiety (Schmidt and Duman, 2010). BDNF is widely discussed as factor altering emotional states including anxiety and depression (for a detailed review see Chourbaji et al., 2011). We analyzed BDNF mRNA levels and could indeed find a reduction in the frontal cortex, but unaltered levels in the hippocampus. In previous studies, we could show that a heterozygous deletion of BDNF was not *per se* responsible for changed emotional states (Chourbaji et al., 2004) but rather dependent on gene-environment interactions like different housing conditions in combination with the heterozygous deletion (Chourbaji et al., 2008). In general, the findings of BNDF levels affecting the emotional state is diverse (for a detailed review see Chourbaji et al., 2011) with positive or negative findings concerning the role of BDNF on anxiety dependent on the generated mouse lines (Monteggia et al., 2007; Autry et al., 2009). Keeping this in mind, the slightly reduced BDNF levels in the frontal cortex only could be a consequence of the *Creb* ablation but are an insufficient explanation for the altered anxiety levels seen in the *Creb* mutants analyzed here.

Another assumption could be that CREB changes (possibly via altered BDNF levels) neurogenesis or neurodegeneration (Mantamadiotis et al., 2002). Our data did not show altered proliferation in the mice; however our mice were over 5 months old, so proliferation rate after one BrdU injection was already quite low. In a study of (Li et al., 2009), the phosphodiesterase 4 inhibitor rolipram increased neurogenesis, pCREB levels and surprisingly decreased anxiety levels, an effect which could be completely stopped by Methylazoxymethanol (MAM) treatment, which blocks proliferation. This, additionally to the study of Gundersen et al. (2013), where virus-induced ablation in the hippocampus led to severely increased neurogenesis, could be a hint that neurogenesis is causative for the alterations although we could not show a correlation in these mice at a single time point.

CREB deficiency was often accompanied by adaptive up regulation of the Cre modulator CREM (Hummler et al., 1994; Gundersen et al., 2013), being in line, *Crem* knockout mice demonstrated reduced anxiety (Maldonado et al., 1999). To overcome adaptive effects of CREB/CREM up or down regulation, some studies used knockout strategies by deleting both *Creb* and *Crem* (Mantamadiotis et al., 2002); if effects of *Creb* knockout were therefore driven by adaptive *Crem* regulations could not be excluded, because both factors were absent. In our mice, we could detect a strong up regulation of CREM in both hippocampus and frontal cortex. Mice with a knockout of *Crem* show low anxiety levels in several anxiety tests like the elevated Plus Maze, Open Field or elevated Zero Maze (Maldonado et al., 1999). This altered emotional phenotype was accompanied by a severe hyperactivity (Maldonado et al., 1999), a behavioral feature which we did not discover in the mice used in this study. Due to the high expression of *Crem* mRNA, it could be assumed that these levels may contribute to behavioral consequences as the altered emotional state found here. The missing activity/locomotor changes may be explained by a lack of developmental effects due to the late onset of the *Creb* ablation. ATF-1, the third player besides CREB and CREM in the transcriptional unit stated by Pittenger et al. (2002) was up or down regulated in the hippocampus or frontal cortex. This may not completely exclude the possibility of a role of ATF-1 but the possibility of playing a leading role is rather small.

In conclusion, there are several plausible mechanisms which can explain the anxious phenotype of Crebflox/flox^CamKCreERT2^ mice. Future studies will have to clarify the molecular downstream mechanisms responsible for the phenotype seen here.

As *Creb* is known to play a role in synaptic plasticity, learning and memory, we analyzed hippocampus-dependent spatial reference memory by conducting a Morris water maze (Vogt et al., 2008). Both acquisition of the task and memory retrieval was not impaired in Crebflox/flox^CamKCreERT2^ mice.

In previous studies, various *Creb* mutant mice displayed deficits in both acquisition and spatial reference memory of the water maze task (Bourtchuladze et al., 1994; Gass et al., 1998; Pittenger et al., 2002; Balschun et al., 2003). *Crebα*Δ knock-outs exhibited profound memory deficits (Bourtchuladze et al., 1994), which depended strongly, however, on the genetic background (Gass et al., 1998; Graves et al., 2002). *Creb*^NesCre^ mice also showed strong impairment in memory acquisition and retrieval, while mice with ablation later in development and restricted to the forebrain (*Creb*^CamKCre7^ mice) did not exhibit alterations during acquisition or probe trial (Balschun et al., 2003). This suggests that early induction of *Creb* ablation leads to developmental deficits that cannot be compensated in adulthood.

To further determine the role of CREB during adulthood in hippocampus-dependent associative learning, we exposed Crebflox/flox^CamKCreERT2^ mice to a foot shock-context conditioning procedure and tested the consolidation into long-term memory. To exclude alterations in pain perception as confounding factors, a hotplate test was performed, revealing an unaltered nociception of *Creb* mutants. In previous studies fear conditioning paradigms have demonstrated robust deficits in several *Creb* mutants (Bourtchuladze et al., 1994; Gass et al., 1998; Graves et al., 2002). When placed into the same context 24 h after conditioning, Crebflox/flox^CamKCreERT2^ mice revealed the same level of memory performance as controls.

Similar to the water maze task, the extent of memory deficits in *Creb*αΔ knock-out mice in contextual fear conditioning was dependent on the genetic background (Bourtchuladze et al., 1994; Gass et al., 1998; Graves et al., 2002). In contrast, neither *Creb*^NesCre^ nor *Creb*^CamKCre7^ mice were impaired in contextual fear conditioning (Balschun et al., 2003). However, the tamoxifen-inducible expression of a dominant negative CREB repressor 6–12 h before conditioning impaired memory consolidation during adulthood (Kida et al., 2002). The present study demonstrates that the loss of *Creb* during adulthood can be fully compensated when the ablation is not immediately induced before the conditioning procedure. A re-balanced network could be the reason that we could not find alterations as recent studies showed that the network state during learning, e.g., the level of CREB in single neurons of the lateral amygdala, enhances the possibility of the cell to be part of a neuronal ensemble encoding fear memory (Kim et al., 2013).

Up to date, it had not been possible to breed at least one of the different *Creb* mutant mouse lines on an isogenic background. Therefore, all *Creb*αΔ knock-out mice investigated had been on different, sometimes randomly mixed backgrounds (Bourtchuladze et al., 1994; Gass et al., 1998; Graves et al., 2002; Briand and Blendy, 2013). Similarly, *Creb*^NesCre^ and *Creb*^CamKCre7^ mice were on a randomly mixed C57BL/6 and 129SvEv background (Balschun et al., 2003), while the tamoxifen-inducible repressor mice were bred as F1 generation of C57BL/6 and C3H (Kida et al., 2002) or of C57BL/6 and129 mice (Graves et al., 2002). Notably, the mice used in our study allow for the first time an induced *Creb* ablation in a pure inbred strain regularly used for behavioral analyses.

The behavioral data of the anxiety tests and two gold standard tests assessing hippocampal learning and memory have demonstrated that the induction of *Creb* ablation during adulthood does impair neither spatial reference memory nor context dependent conditioning, but highly impairs emotional behavior. It can be stated that the induced disturbances of CREB pathways can lead to behavioral and molecular consequences. In humans, this relation towards emotional regulation seems also to be relevant as stated by Kerner et al. (2013) linking rare genomic variants of patients with bipolar disorder and co-morbid anxiety as well as panic disorders (Domschke et al., 2003) to CREB signaling pathways.

## Author contributions

Miriam A. Vogt, Dragos Inta, Hasan Elkin and Peter Gass conceived this work, Miriam A. Vogt, Dragos Inta, Hasan Elkin, Natascha Pfeiffer and Peter Gass designed it, Miriam A. Vogt, Dragos Inta, Natascha Pfeiffer and Alessia Luoni acquired data, Miriam A. Vogt, Dragos Inta, Natascha Pfeiffer and Alessia Luoni analyzed data and interpreted data together with Hasan Elkin, Peter Gass and Marco A. Riva. Miriam A. Vogt and Dragos Inta drafted the work; all authors critically revised it and approved the final version.

## Conflict of interest statement

The authors declare that the research was conducted in the absence of any commercial or financial relationships that could be construed as a potential conflict of interest.
